# Usage of Plant Food Supplements across Six European Countries: Findings from the PlantLIBRA Consumer Survey

**DOI:** 10.1371/journal.pone.0092265

**Published:** 2014-03-18

**Authors:** Alicia Garcia-Alvarez, Bernadette Egan, Simone de Klein, Lorena Dima, Franco M. Maggi, Merja Isoniemi, Lourdes Ribas-Barba, Monique M. Raats, Eva Melanie Meissner, Mihaela Badea, Flavia Bruno, Maija Salmenhaara, Raimon Milà-Villarroel, Viktoria Knaze, Charo Hodgkins, Angela Marculescu, Liisa Uusitalo, Patrizia Restani, Lluís Serra-Majem

**Affiliations:** 1 Fundación para la Investigación Nutricional, Barcelona Science Park, University of Barcelona, Barcelona, Spain; 2 Food, Consumer Behaviour and Health Research Centre, University of Surrey, Guildford, Surrey, United Kingdom; 3 PhytoLab GmbH & Co KG, Vestenbergsgreuth, Germany; 4 Transilvania University of Brasov, Brasov, Romania; 5 Dipartimento di Scienze Farmacologiche e Biomolecolari, Università degli Studi di Milano, Milano, Italy; 6 Finnish Food Safety Authority Evira, Helsinki, Finland; 7 Ciber Obn Fisiopatología de la Obesidad y la Nutrición, Instituto de Salud Carlos III, Madrid, Spain; 8 Institute of Biomedical and Health Research of Las Palmas, University of Las Palmas de Gran Canaria, Las Palmas de Gran Canaria, Spain; University of East Anglia, United Kingdom

## Abstract

**Background:**

The popularity of botanical products is on the rise in Europe, with consumers using them to complement their diets or to maintain health, and products are taken in many different forms (e.g. teas, juices, herbal medicinal products, plant food supplements (PFS)). However there is a scarcity of data on the usage of such products at European level.

**Objective:**

To provide an overview of the characteristics and usage patterns of PFS consumers in six European countries.

**Design:**

Data on PFS usage were collected in a cross-sectional, retrospective survey of PFS consumers using a bespoke frequency of PFS usage questionnaire.

**Subjects/setting:**

A total sample of 2359 adult PFS consumers from Finland, Germany, Italy, Romania, Spain and the United Kingdom.

**Data analyses:**

Descriptive analyses were conducted, with all data stratified by gender, age, and country. Absolute frequencies, percentages and 95% confidence intervals are reported.

**Results:**

Overall, an estimated 18.8% of screened survey respondents used at least one PFS. Characteristics of PFS consumers included being older, well-educated, never having smoked and self-reporting health status as “good or very good”. Across countries, 491 different botanicals were identified in the PFS products used, with *Ginkgo biloba* (Ginkgo), *Oenothera biennis* (Evening primrose) and *Cynara scolymus* (Artichoke) being most frequently reported; the most popular dose forms were capsules and pills/tablets. Most consumers used one product and half of all users took single-botanical products. Some results varied across countries.

**Conclusions:**

The PlantLIBRA consumer survey is unique in reporting on usage patterns of PFS consumers in six European countries. The survey highlights the complexity of measuring the intake of such products, particularly at pan-European level. Incorporating measures of the intake of botanicals in national dietary surveys would provide much-needed data for comprehensive risk and benefit assessments at the European level.

## Introduction

Botanicals and their derivatives/preparations are used throughout Europe for health purposes, with increased usage in the general population as well as among specific subgroups encompassing children and pregnant women or those suffering from diseases such as cancer among others [1]–[4]. Botanicals are used in many different types of products, including foods, (teas and juices), food supplements such as plant food supplements (PFS), herbal medicinal products (HMP), homeopathic products, cosmetics, biocides etc [5]. These different product categories are regulated by specific legislation, depending on the intended use of the product.

The European Union (EU) Directive on Food Supplements (2002/46/EC) defines dietary supplements (which include PFS) as [6]:

“…foodstuffs the purpose of which is to supplement the normal diet and which are concentrated sources of nutrients or other substances with a nutritional or physiological effect, alone or in combination, marketed in dose form, namely forms such as capsules, pastilles, tablets, pills and other similar forms, sachets of powder, ampoules of liquids, drop dispensing bottles and other similar forms of liquids and powders designed to be taken in measured small quantities”.

The marketing of a product as a PFS however, depends on national legislation, which differs widely across Member States. Countries vary in the extent to which products are regulated, as well as in the process of regulatory control. Some countries have regulated the use of botanicals in detail (including negative and positive lists), some apply specific conditions of use, (including maximum usage levels or warnings for the consumer), and in others less specific requirements exist. An added complexity lies in the application of the basic European “principle of mutual recognition”, whereby any product that is lawfully marketed in one Member State can be sold in all 27 Member States [5].

Moreover, the same botanical may be used as a food supplement and as a medicinal product, depending on the intended use of the product and both food supplements and medicinal products often share the same form of presentation (powders, pills or tablets). Hence the legal status of products differs from one country to another, resulting in a complex market environment. This so-called borderline issue between PFS and HMP is a major obstacle to the marketing of PFS in the European Union [5].

Plant food supplement usage data at EU level are scarce with reports providing PFS market data as opposed to data reported directly by the consumer [7]. Surveys on the intake of botanicals have been conducted primarily in the context of the intake of dietary supplements in general [8] or as part of surveys of complementary and alternative medicine (CAM) therapies [9], and issues such as the legal distinction between HMP and PFS have not been taken into account. A recent systematic review evaluating the demographic characteristics and health status factors associated with CAM use reported that the majority of population based consumption studies had been conducted in the USA (64% of the 110 identified studies), and of these, 13% were in Europe, with the majority carried out in Scandinavia (7%) and the United Kingdom (5%) [4]. Studies have been limited by the heterogeneity of definitions used, study designs and objectives making it difficult to compare results and to extrapolate conclusions. The ambiguity of categories such as “natural medicine”, “herbal remedies” or “herbal medicine” and what constitutes “dietary supplements” makes it nearly impossible to attain reliable estimates of the prevalence of PFS usage in Europe, with only limited data available at national levels [9]–[11] but not at the European level.

A study by the European Advisory Services (EAS) on “The use of substances with nutritional or physiological effect other than vitamins and minerals in food supplements” [7], provided information on European market and regulation data, and highlighted the need for obtaining PFS usage data in order to plan, monitor and evaluate national and European policies, as in other regions of the world. One such example is the United States of America, where the Alternative Health/CAM supplement of the National Health Interview Survey (NHIS) has been collecting data on botanical dietary supplements for some years now [12]–[14].

The European Food Safety Authority (EFSA) has recognised the lack of data in the sector and has published a number of reports addressing related issues, namely the recommendations for reporting the use of supplements and medicines by adults in any pan-European dietary survey or project [15], and the “Compendium of botanicals reported to contain naturally occurring substances of possible concern for human health”, aimed to help with the safety assessment of botanicals and botanical preparations intended for use as food supplements [16].

The purpose of this paper is to describe the type and frequency of PFS usage reported in a retrospective survey of consumers in six European countries; in addition we present the most frequently used botanical ingredients in these products. We also highlight the issues associated with measuring usage of PFS in European populations and make recommendations for future research.

## Materials and Methods

### Ethics statement

Before initiating the fieldwork, approval for the conduct of the survey was obtained from four ethics committees: the Bioethics Commission of the University of Barcelona, Spain; the Ethics Committee of the University of Milano, Italy; the Ethical Committee of the Faculty of Medicine - Transilvania University of Brasov, Romania; and the Coordinating Ethics Committee, Hospital District of Helsinki and Uusimaa, Finland. Approval of the survey by these four ethics committees required submitting all survey material to their members for evaluation. No ethical approval for the survey was needed in Germany and the United Kingdom.

To ensure harmonisation and standardisation of the fieldwork and data collection across countries, a market research organization, *European Fieldwork Group* (EFG) was subcontracted to implement the survey. The survey was conducted by EFG in strict accordance with the ICC/ESOMAR Code on Market and Social Research. In all countries, informed consent was obtained verbally from all respondents after reading the survey information sheet. All data were recorded manually i.e. pen-and-paper. Recruitment of survey participants occurred in the selected cities in each country. Approximately the first 1000 individuals per country were systematically selected for screening i.e. intercepting 1 in every 5 individuals passing by to ask him/her the initial screening questions; subsequent screening selection was performed on a convenience basis i.e. intercepting individuals in places where consumers were likely to be found, such as herbal shops, pharmacies etc. Eligible respondents who agreed to participate were given an appointment at their home/workplace to complete the main survey. The appointments of those willing to participate were later reconfirmed by phone.

The data were made anonymous when recorded electronically i.e. the respondents' contact details were not entered into the survey database. Instead, the market research organization assigned ID numbers to each respondent and provided PlantLIBRA partners only the database with the assigned ID numbers.

### Definition of plant food supplements in the PlantLIBRA PFS consumer survey

Although there is a legal definition of Food Supplements (EU Directive (2002/46/EC) [6] under which PFS reside, for the purposes of this research it was necessary to develop a specific definition of PFS whose main characteristic is that they contain botanical preparations as ingredients for food supplementation.

Botanical preparations are obtained by subjecting botanicals (plants, algae, fungi or lichens) to treatments such as comminution, extraction, distillation, squeezing, fractionation, purification, concentration or fermentation. These include extracts, essential oils, expressed juices, powders, etc.

Botanical preparations can be considered as *nutrients* or *other substances*. Thus, the definition of PFS for the survey was as follows: PFS are "foodstuffs the purpose of which is to supplement the normal diet and which are concentrated sources of botanical preparations that have nutritional or physiological effect, alone or in combination with vitamins, minerals and other substances which are not plant-based. PFS are marketed in dose form, such as capsules, pastilles, tablets, pills and other similar forms, sachets of powder, ampoules of liquids, drop dispensing bottles, and other similar forms of liquids and powders designed to be taken in measured small unit quantities”.

Products that did not meet this definition, such as herbal remedies and other medicinal products based on botanicals, and those that did not meet the PFS definition in terms of dosage, such as herbal teas or juices, were excluded.

### Sample population and PFS consumer definition

A cross-sectional, 12-month retrospective survey was conducted in 24 cities in six European countries -Finland, Germany, Italy, Romania, Spain and the United Kingdom. An estimated sample size of 2000 screened individuals per country was calculated in order to obtain a final sample of approximately 400 consumers per country (total N = 2400 approximately). Per country, gender and age group quotas were set as follows: 300 adults (18 to 59 years) and 100 older adults (60-and-over years), with 30–50% male and 50–70% female. All individuals were screened by means of a brief questionnaire which recorded PFS usage in the preceding 12 months. Individuals were considered eligible for inclusion if they were over 18 years old and met either of the following specified criteria, intended to capture the different usage patterns of PFS consumers:

1. They had taken at least 1 PFS in the last 12 months, in an appropriate dose form at a minimum frequency of either:
  - 1 daily dose for at least 2 consecutive or non-consecutive weeks, or
  - 1 or more doses per week for at least 3 consecutive weeks or
  - 1 or more doses per week for at least 4 consecutive or non-consecutive weeks
2. They had taken 2 or more different PFS, in an appropriate dose form, at a minimum frequency of 1 or more doses per week, with the sum of the usage period of the 2 or more products being equal to at least 4 weeks.

### Instruments and variables

A short screening questionnaire was used to identify consumers who met the survey inclusion criteria; it consisted of six questions which allowed interviewers to identify eligible consumers, based on the product(s) used, the frequency and duration of use and the dose form. Eligible consumers subsequently completed a more detailed questionnaire on their PFS usage in the preceding 12 months, providing details of product/plant names, dosage forms, frequency of use, reasons for use, adverse effects, places and patterns of purchase and information sources on products. These questions were asked for each of up to a maximum of 5 different PFS used. In addition, respondents were asked to provide socio-demographic data including age, gender, level of education and employment status, as well as self-reported height and weight and further health-related lifestyle information.

### Survey administration and data collection

Fieldwork and data collection for the cross-sectional survey were conducted by the international market research company EFG, from May 2011 to September 2012. The duration of the fieldwork ensured that any seasonal variability in usage of products was captured. The survey protocols and instruments -training material, information sheet, informed consent, screening and usage questionnaires-, were initially developed in English by consensus amongst the research team, and subsequently translated into the respective languages in each of the survey countries. Pilot interviews were conducted in each participating country to assess the comprehension of the questions and to determine the time required to complete the survey.

In each participating country, trained interviewers systematically screened approximately 1000 individuals during the first three months of the survey, which allowed the estimation of the prevalence rate. Subsequently, screening and recruitment were conducted on a convenience basis. The recruited eligible consumers were interviewed face-to-face and the more detailed PFS usage questionnaire completed.

### Data preparation and statistical analysis

All data from the completed surveys were entered into the statistical package SPSS for Windows v. 18 (IBM Corporation, Somers, NY, USA), which was also used for data analysis.

Following review of the completed interviews by the research team in each country, a database with botanical composition data for all PFS products reported was compiled for each country and then merged into a single database. Potential product duplicates between countries were not removed. Each product was coded for its botanical ingredients in scientific, English and local names and botanicals were coded after removing duplicates between countries. Additionally, each product was categorised as a single- or multi-botanical product. To indicate the certainty of the matching of products, a series of numerical codes were used, based on those used in the National Health and Nutrition Examination Survey 2005–2006 [17]. Values ranged from 1–5, where “1” indicated an exact match, “2” a probable match, “3” a reasonable match, “4” a default match and “5” no match. Only products with certainty values 1 to 4 have been included in the analyses.

Respondent data were recorded in a separate database. A number of variables were created and/or recoded to facilitate reporting and analysis, including: 1) “education level”, defined as low, medium, and high; 2) “BMI”, which was calculated from self-reported weight and height, and for which WHO criteria [18] were used to categorise individuals as underweight (BMI<18.5 kg/m^2^), normal weight (BMI 18.5-<25 kg/m^2^), overweight (BMI 25-<30 kg/m^2)^ and obese (BMI ≥30 kg/m^2^); 3) “physical activity”, calculated using the short version of the IPAQ [19] and defined as low, moderate or high.

Absolute frequencies and percentages for each of the variable categories were used to describe the qualitative nominal/ordinal and discrete quantitative survey data. In turn, all data have been stratified by gender, age range and country - also using absolute frequencies and percentages and 95% confidence intervals. When describing the association between two qualitative variables (nominal or ordinal), contingency tables were used. The continuous quantitative variables (e.g. BMI, alcohol) were recoded into categorical variables.

It is important to note that when reporting the main results of the survey, the unit of analysis varies depending on the variables used, i.e. for certain variables the unit is an individual respondent, however, given the potential intake of multiple supplements by one respondent, the unit of analysis may change to the supplement level. Furthermore, all results presented in the tables represent the analysis of raw data as opposed to data weighted by the population size. Data were not weighted because of the study methodology selected, whereby all country samples were very similar in size and included only PFS consumers.

### Validation study

In order to validate the PFS usage questionnaire, a validation study was conducted in which the data collected using the survey instrument were compared with a 30 to 180-day diary (used as the gold standard). The study was conducted in two of the PlantLIBRA consumer survey cities: Las Palmas de Gran Canaria (Spain) and Milan (Italy), where 48 and 49 consumers respectively were recruited using convenience sampling. The PFS usage questionnaire was completed by the respondents at the beginning and at the end of the 6-month period of the validation; during this time the consumers also completed the usage diary. Data from the last questionnaire and the diary were compared for concordance, and results are shown in Table 1, indicating a good agreement for product consumed, dose form and doses per day.

**Table 1:** Validation study results.

Variable	Concordance^a^	Milan		Las Palmas de Gran Canaria	
		n	%	n	%
Product used	Yes	47	95.9	48	100.0
	No	2	4.1	0	0.0
Dose form (pills, capsules, etc)	Yes	45	91.8	47	97.9
	No	4	8.2	1	2.1
Doses per day	Yes	45	91.8	38	79.2
	No	4	8.2	10	20.8

a Concordance between both methods: the PFS usage questionnaire and the 6-month usage diary.

## Results

### Characteristics of the PFS consumer sample

A final sample of 2359 consumers (those eligible and willing to participate) was recruited from 11783 screened individuals (Table 2). Due to different legal frameworks (different distribution of botanicals in food supplements and medicinal products), more individuals had to be screened in Finland in order to recruit the required 400 consumers. Table 2 also shows the sample used for the estimation of the usage prevalence rate. The estimated weighted overall PFS usage prevalence rate was 18.8% and per-country rates were as follows: Finland 9.6%, Germany 16.9%, Italy 22.7%, Romania 17.6%, Spain 18.0% and the United Kingdom 19.1%.

**Table 2:** Distribution of screened individuals, PFS consumers interviewed and prevalence sample by country and gender.

			Finland	Germany	Italy	Romania	Spain	United Kingdom	Total
Total contacts (n)	Total individuals screened for the survey	Males	1405	1031	907	795	811	830	5779
		Females	1379	1028	1044	827	932	794	6004
	Total PFS consumers interviewed accepted	Males	193	197	187	199	174	191	1141
		Females	208	201	191	201	228	189	1218
Prevalence sample: systematically selected sample 1st three months of the Fieldwork (n)	Individuals screened	Males	486	564	439	502	551	454	2996
		Females	519	571	547	501	648	563	3349
	PFS consumers among Individuals screened	Males	33	90	99	95	55	65	437
		Females	71	111	156	124	133	144	739
PFS consumption prevalence (weighted) (%)			9.6	16.9	22.7	17.6	18.0	19.1	18.8

Survey respondents were recruited to fixed quotas for age and gender, which were achieved, with some differences within countries (Table 3). In Finland the proportion of adults aged 50–59 years was significantly higher (26.2%), whilst the opposite was true in Italy, where consumers in that age group constituted only 13.0% of adults. Romania had a significantly higher number of consumers in the youngest age group (30.5%), in contrast to Spain and the United Kingdom, where this age group represented only 9.5% and 9.0% of adult consumers, respectively. A significantly higher proportion of female consumers were recruited in Spain (56.7%) and in the United Kingdom marginally more males were recruited (50.3%). Across all countries, more than half of the participants (57.5%) were employed (Table 3), with the percentages slightly lower in Finland (50.9%) and in the United Kingdom (52.4%). The majority of participating consumers were educated to medium level (Table 3).

**Table 3:** PlantLIBRA's PFS consumer survey – socio-demographic sample characteristics, overall and by country.

Characteristics	Categories	All countries		Finland		Germany		Italy		Romania		Spain		United Kingdom	
		n	% (95% CI)	n	% (95% CI)	n	% (95% CI)	n	% (95% CI)	n	% (95% CI)	n	% (95% CI)	n	% (95% CI)
Gender	Male	1141	48.4 (46.4–50.4)	193	48.1 (43.2–53.0)	197	49.5 (44.6–54.4)	187	49.5 (44.4–54.5)	199	49.8 (44.8–54.7)	174	43.3 (38.4–48.1)	191	50.3 (45.2–55.3)
	Female	1218	51.6 (49.6–53.7)	208	51.9 (47.0–56.8)	201	50.5 (45.6–55.4)	191	50.5 (45.5–55.6)	201	50.3 (45.3–55.2)	228	56.7 (51.9–61.6)	189	49.7 (44.7–54.8)
Age	18–29 years	418	17.7 (16.2–19.3)	63	15.7 (12.1–19.3)	77	19.4 (15.5–23.2)	84	22.2 (18.0–26.4)	122	30.5 (26.0–35.0)	38	9.5 (6.6–12.3)	34	9.0 (6.1–11.8)
	30–39 years	445	18.9 (17.3–20.4)	65	16.2 (12.6–19.8)	57	14.3 (10.9–17.8)	88	23.3 (19.0-27.6)	65	16.3 (12.6–20.0)	101	25.1 (20.9–29.4)	69	18.2 (14.3–22.0)
	40–49 years	460	19.5 (17.9–21.1)	64	16.0 (12.4–19.6)	82	20.6 (16.6–24.6)	63	16.7 (12.9–20.4)	46	11.5 (8.4–14.6)	88	21.9 (17.8–25.9)	117	30.8 (26.1–35.4)
	50–59 years	441	18.7 (17.1–20.3)	105	26.2 (21.9–30.5)	80	20.1 (16.2–24.0)	49	13.0 (9.6–16.4)	67	16.8 (13.1–20.4)	76	18.9 (15.1–22.7)	64	16.8 (13.1–20.6)
	≥60 years	595	25.2 (23.5–27.0)	104	25.9 (21.6–30.2)	102	25.6 (21.3–29.9)	94	24.9 (20.5–29.2)	100	25.0 (20.8–29.3)	99	24.6 (20.4–28.8)	96	25.3 (20.9–29.6)
Education	Low	249	10.6 (9.3–11.8)	47	11.7 (8.6–14.9)	3	0.8 (0.0–1.6)	72	19.1 (15.1–23.0)	35	8.8 (6.0–11.5)	92	22.9 (18.8–27.0)	0	–
	Medium	1549	65.7 (63.6–67.6)	237	59.1 (54.3–63.9)	329	82.7 (78.9–86.4)	222	58.7 (53.8–63.7)	190	47.5 (42.6–52.4)	256	63.7 (59.0–68.4)	315	82.9 (79.1–86.7)
	High	561	23.8 (22.1–25.5)	117	29.2 (24.7–33.6)	66	16.6 (12.9–20.2)	84	22.2 (18.0–26.4)	175	43.8 (38.9–48.6)	54	13.4 (10.1–16.8)	65	17.1 (13.3–20.9)
Current employment status	Employed	1357	57.5 (55.5–59.5)	204	50.9 (46.0–55.8)	240	60.3 (55.5–65.1)	221	58.5 (53.5–63.4)	249	62.3 (57.5–67.0)	244	60.7 (55.9–65.5)	199	52.4 (47.3–57.4)
	Other groups a	1002	42.5 (40.9–44.5)	197	49.1 (44.2–54.0)	158	39.7 (34.9–44.5)	157	41.5 (36.6–46.5)	151	37.8 (33.0–42.5)	181	39.3 (34.5–44.1)	181	47.6 (42.6–52.7)

a Other groups: Unemployed; Housework; Student; Retired; Disabled; and Other.

Respondents were asked a number of questions regarding health-related lifestyle factors (Table 4). Less than half of the consumers had never smoked (46.6%), less than one quarter were ex-smokers (23.1%) and less than one third were current smokers (30.3%).

**Table 4:** PlantLIBRA's PFS consumer survey – health-related lifestyle sample characteristics, overall and by country.

Characteristics	Categories	All countries		Finland		Germany		Italy		Romania		Spain		United Kingdom	
		n	% (95% CI)	n	% (95% CI)	n	% (95% CI)	n	% (95% CI)	n	% (95% CI)	n	% (95% CI)	n	% (95% CI)
Regular use of non-PFS FS^ab^	No	1536	65.1 (63.2–67.0)	83	20.7 (16.7–24.7)	251	63.1 (58.3–67.8)	311	82.3 (78.4–86.1)	274	68.5 (63.9–73.1)	312	77.6 (73.5–81.7)	305	80.3 (76.3–84.3)
	Yes	767	32.5 (30.6–34.4)	306	76.3 (72.1–80.5)	122	30.7 (26.1–35.2)	63	16.7 (12.9–20.4)	112	28.0 (23.6–32.4)	89	22.1 (18.1–26.2)	75	19.7 (15.7–23.7)
	Not sure	56	2.4 (1.8–3.0)	12	3.0 (1.3–4.7)	25	6.3 (3.9–8.7)	4	1.1 (0.1–2.1)	14	3.5 (1.7–5.3)	1	0.3 (0.0–0.7)	0	–
Smoking habit	Never smoker	1100	46.6 (44.6–48.6)	182	45.4 (40.5–50.3)	183	46.0 (41.1–50.9)	181	47.9 (42.8–52.9)	214	53.5 (48.6–58.4)	177	44.0 (39.2–48.9)	163	42.9 (37.9–47.9)
	Former smoker	544	23.1 (21.4–24.8)	129	32.2 (27.6–36.8)	81	20.4 (16.4–24.3)	85	22.5 (18.3–26.7)	57	14.3 (10.8–17.7)	94	23.4 (19.2–27.5)	98	25.8 (21.4–30.2)
	Current smoker	715	30.3 (28.5–32.2)	90	22.4 (18.4–26.5)	134	33.7 (29.0–38.3)	112	29.6 (25.0–34.2)	129	32.3 (27.7–36.8)	131	32.6 (28.0–37.2)	119	31.3 (26.7–36.0)
Self-reported health status	Very good	353	15.0 (13.5–16.4)	81	20.2 (16.3–24.1)	49	12.3 (9.1–15.5)	22	5.8 (3.5–8.2)	80	20.0 (16.1–23.9)	49	12.2 (9.0–15.4)	72	19.0 (15.0–22.9)
	Good	1427	60.5 (58.5–62.5)	225	56.1 (51.3–61.0)	220	55.3 (50.4–60.2)	243	64.3 (59.5–69.1)	245	61.3 (56.5–66.0)	258	64.2 (59.5–68.9)	236	62.1 (57.2–67.0)
	Neither bad nor good	496	21.0 (19.4–22.7)	77	19.2 (15.3–23.1)	111	27.9 (23.5–32.3)	111	29.4 (24.8–34.0)	73	18.3 (14.5–22.0)	81	20.2 (16.2–24.1)	43	11.3 (8.1–14.5)
	Bad	70	3.0 (2.3–3.7)	16	4.0 (2.1–5.9)	18	4.5 (2.5–6.6)	2	0.5 (0.0–1.3)	2	0.5 (0.0–1.2)	14	3.5 (1.7–5.3)	18	4.7 (2.6–6.9)
	Very bad	13	0.6 (0.3–0.9)	2	0.5 (0.0 – 1.2)	0	–	0	–	0	–	0	–	11	2.9 (1.2–4.6)
CAM^c^ usage	Yes	947	40.1 (38.2–42.1)	223	55.6 (50.7–60.5)	204	51.3 (46.3–56.2)	96	25.4 (21.0–29.8)	77	19.3 (15.4–23.1)	319	79.4 (75.4–83.3)	28	7.4 (4.7–10.0)
	No	1412	59.9 (57.9–61.8)	178	44.4 (39.5–49.3)	194	48.7 (43.8–53.7)	282	74.6 (70.2–79.0)	323	80.8 (76.9–84.6)	83	20.7 (16.7–24.6)	352	92.6 (90.0–95.3)
Alcohol consumption	0-<1 times/day	1398	59.3 (57.3–61.3)	281	70.1 (65.6–74.6)	245	61.6 (56.8–66.3)	116	30.7 (26.0–35.3)	232	58.0 (53.2–62.8)	291	72.4 (68.0–76.8)	233	61.3 (56.4–66.2)
	≥1 times/day	296	12.6 (11.2–13.9)	13	3.2 (1.5–5.0)	27	6.8 (4.3–9.3)	156	41.3 (36.3–46.2)	9	2.3 (0.8–3.7)	46	11.4 (8.3–14.6)	45	11.8 (8.6–15.1)
	Not sure	614	26.0 (24.3–27.8)	107	26.7 (22.4–31.0)	126	31.7 (27.1–36.2)	106	28.0 (23.5–32.6)	159	39.8 (35.0–44.6)	65	16.2 (12.6–19.8)	102	26.8 (22.4–31.3)
BMI^d^ categories	Underweight	69	2.9 (2.4–3.6)	9	2.2 (0.8–3.7)	4	1.0 (0.0–2.0)	12	3.2 (1.4–4.9)	20	5.0 (2.9–7.1)	6	1.5 (.3–2.7)	18	4.7 (2.6–6.9)
	Normal weight	1116	47.3 (45.3–49.3)	188	46.9 (42.0–51.8)	198	49.7 (44.8–54.7)	246	65.1 (60.3–69.9)	184	46.0 (41.1–50.9)	169	42.0 (37.2–46.9)	131	34.5 (29.7–39.3)
	Overweight	818	34.7 (32.8–36.6)	147	36.7 (31.9–41.4)	159	40.0 (35.1–44.8)	98	25.9 (21.5–30.4)	142	35.5 (30.8–40.2)	155	38.6 (33.8–43.3)	117	30.8 (26.1–35.4)
	Obesity	356	15.1 (13.7–16.5)	57	14.2 (10.8–17.6)	37	9.3 (6.4–12.2)	22	5.8 (3.5–8.2)	54	13.5 (10.2–16.9)	72	17.9 (14.2–21.7)	114	30.0 (25.4–34.6)
Physical activity^e^	Low	436	18.5 (16.9–20.1)	53	13.2 (9.9–16.5)	87	21.9 (17.8–25.9)	141	37.3 (32.4–42.2)	5	1.3 (0.2–2.3)	43	10.7 (7.7–13.7)	107	28.2 (23.6–32.7)
	Moderate	909	38.5 (36.6–40.5)	156	38.9 (34.1–43.7)	139	34.9 (30.2–39.6)	191	50.5 (45.5–55.6)	53	13.3 (9.9–16.6)	234	58.2 (53.4–63.0)	136	35.8 (31.0–40.6)
	High	1012	42.9 (40.9–44.9)	192	47.9 (43.0–52.8)	171	43.0 (38.1–47.8)	45	11.9 (8.6–15.2)	342	85.5 (82.1–89.0)	125	31.1 (26.6–35.6)	137	36.1 (31.2–40.9)

a ***Question asked*** **:** Other than PLANT FOOD SUPPLEMENT, have you taken any of the following supplements on a regular basis in the last 12 months? (mark all that apply). ***Possible responses***: Vitamins (A, B, D, E, etc.); Minerals (eg. potassium, calcium); Amino acids; Enzymes (eg. lactase); Prebiotics (eg. oligosaccharides, fibre); Probiotics (eg. bifidobacteria, yeasts); Fatty acids (eg. fish oil); Other.

b FS =  Food supplements.

c CAM = Complementary and Alternative Medicine, including: Acupuncturist; Chiropractor; Homeopath; Herbalist; Massage therapist; Traditional/faith healer; Reflexologist; Recognised treatment i.e. not "alternative"; Esoteric treatment; and “Cannot be classified”.

d BMI =  Body Mass Index; WHO categories [18].

e IPAQ categories [19].

More than half of the total respondents (59.3%) had not consumed alcohol or had consumed it less than once daily; more than a tenth (12.6%) reported daily alcohol consumption.

The proportion of overweight and obese people in the survey was 49.8% (Table 4). Some significant differences in levels of physical activity were noted between countries. High levels of activity were reported by 85.5% of Romanian respondents compared to a value of 42.9% across all countries.

Most of the respondents (65.1%) reported not being regular consumers of food supplements other than PFS in the preceding 12 months, except for Finland (Table 4). The proportion of non-consumers varied from 20.7% in Finland to more than 80% in the United Kingdom and Italy. By contrast, in Finland 76.3% of the individuals were regular consumers of food supplements.

Over half of all respondents (59.5%) reported not having used CAM therapies/treatments in the past year. This is particularly the case in Italy (74.6%), Romania (80.8%) and the United Kingdom (92.6%).

Three quarters of consumers reported their health status as very good or good (75.5%), while 3.6% reported it as bad or very bad and 21.0% as neither bad nor good (Table 4).

Between countries, more consumers reported their health status as very good or good in Romania (81.3%) and in the United Kingdom (81.1%) than in other countries; though conversely the highest proportion reporting their health status as bad or very bad was also in the United Kingdom (7.6%).

### PFS usage patterns

Overall, products are most often taken “periodically” (37.3%) with respondents also reporting using PFS when experiencing a “flare up or worsening of a condition” (22.2%) (Table 5). Products are also used on a more “sporadic basis” (19.8%) and on “other non-specified occasions” (17.8%). Both men and women reported taking products on a periodic basis (39.3%, 35.6%) and this was also true for both age groups (Table 5). Periodic use was reported significantly more often in Finland (46.2%), Germany (50.7%), Italy (41.3%) and Romania (41.8%), but in Spain, “another reason” was most reported (46.0%) and in the United Kingdom, sporadic use (34.8%) was significantly higher than any other reason as to when products were used (Table 6).

**Table 5:** PlantLIBRA's PFS consumer survey – PFS usage patterns, per product used by a respondent, overall and by gender and age group.

			Gender				Age group			
	Total (n = 2874)		Male (n = 1358)		Female (n = 1516)		18–59 years (n = 2131)		≥60 years (n = 743)	
	n	% (95% CI)	n	% (95% CI)	n	% (95% CI)	n	% (95% CI)	n	% (95% CI)
I took it whenever/sporadically	568	19.8 (18.3–21.2)	280	20.6 (18.5–22.8)	288	19.0 (17.0–21.0)	437	20.5 (18.8–22.2)	131	17.6 (14.9–20.4)
I take it periodically, during those times only	1072	37.3 (35.5–39.1)	533	39.3 (36.7–41.9)	539	35.6 (33.1–38.0)	827	38.8 (36.7–40.9)	245	33.0 (29.6–36.46)
I took it when I had a flare up/worsening of condition	638	22.2 (20.7–23.7)	278	20.5 (18.3–22.6)	360	23.8 (21.6–25.9)	451	21.2 (19.4–22.9)	187	25.2 (22.1–28.3)
Other reason	512	17.8 (16.4–19.2)	224	16.5 (14.5–18.5)	288	19.0 (17.0–21.0)	353	16.6 (15.0–18.1)	159	21.4 (18.5–24.4)
Not sure	84	2.9 (2.3–3.5)	43	3.2 (2.2–4.1)	41	2.7 (1.9–3.5)	63	3.0 (2.2–3.7)	21	2.8 (1.6–4.0)

***Questions asked.*** During the last 12 months, in what months have you taken this supplement? (mark all that apply). ***Possible responses***: Jan, Feb, Mar, Apr, May, June, July, Aug, Sep, Oct, Nov, Dec, All year round; Why did you decide to take this supplement in the months stated? (one answer only). ***Possible responses***: I took it whenever/sporadically; I take it periodically, during those times only; When I had a flare up/worsening of condition; Other reason; Not sure.

**Table 6:** PlantLIBRA's PFS consumer survey – PFS usage patterns, per product used by a respondent, overall and by country.

	Finland (n = 665)		Germany (n = 446)		Italy (n = 417)		Romania (n = 464)		Spain (n = 465)		United Kingdom (n = 417)	
	n	% (95% CI)	n	% (95% CI)	n	% (95% CI)	n	% (95% CI)	n	% (95% CI)	n	% (95% CI)
I took it whenever/sporadically	83	12.5 (10.0–15.0)	102	22.9 (19.0–26.8)	73	17.5 (13.9–21.2)	60	12.9 (9.9–16.0)	105	22.6 (18.8–26.4)	145	34.8 (30.2–39.4)
I take it periodically, during those times only	307	46.2 (42.4–50.0)	226	50.7 (46.0–55.3)	172	41.3 (36.5–46.0)	194	41.8 (37.3–46.3)	68	14.6 (11.4–17.8)	105	25.2 (21.0–29.4)
I took it when I had a flare up/worsening of condition	126	19.0 (16.0–21.9)	89	20.0 (16.2–23.7)	128	30.7 (26.3–35.1)	117	25.2 (21.3–29.2)	75	16.1 (12.8–19.5)	103	24.7 (20.6–28.8)
Other reason	140	21.1 (18.0–24.2)	26	5.8 (3.7–8.0)	32	7.7 (5.1–10.2)	51	11.0 (8.1–13.8)	214	46.0 (41.5–50.6)	49	11.8 (8.7–14.9)
Not sure	9	1.4 (0.5–2.2)	3	0.7 (0.0–1.4)	12	2.9 (1.3–4.5)	42	9.1 (6.4–11.7)	3	0.7 (0.0–1.4)	15	3.6 (1.8–5.4)

***Questions asked.*** During the last 12 months, in what months have you taken this supplement? (mark all that apply) ***Possible responses***: Jan, Feb, Mar, Apr, May, June, July, Aug, Sep, Oct, Nov, Dec, All year round; Why did you decide to take this supplement in the months stated? (one answer only) ***Possible responses***: I took it whenever/sporadically; I take it periodically, during those times only; When I had a flare up/worsening of condition; Other reason; Not sure.

### PFS products used

Respondents reported a total of 1288 products across the six countries. At individual country level, the highest numbers of different PFS were used in Italy (289) and Spain (284); in the United Kingdom, the number of different PFS was approximately half that of the other countries (Table 7). The number of different botanical ingredients was 491, with the maximum number of different botanicals contained in a single product being 46 and present in a German product. The United Kingdom differed from the other countries as the products reported contained a lower number of botanical ingredients (maximum 8).

**Table 7:** PlantLIBRA's PFS consumer survey – Characteristics of PFS reported by respondents.

	Total	Finland	Germany	Italy	Romania	Spain	United Kingdom
Number of products	1288	213	190	289	196	284	116
Number of botanicals	491	196	191	222	219	218	47
Number of manufacturers	449	69	99	106	61	97	17
Maximum number of ingredients per product	46	23	46	20	39	30	8

In terms of the number of products used, 83.7% of all consumers reported taking one product in the preceding 12 months, with 12.3% taking two products and 4.0% using more than two products (Table 8). Generally this pattern was similar for both men and women and across the age groups, although those over 60 did report a significantly higher use of two or more products than those under 60 (19.5% vs. 15.2%) (Table 8). At country level (Table 9), some significant differences were noted: in Finland, the percentage of consumers using two or more products was significantly higher than in all other countries (40.2%).

**Table 8:** PlantLIBRA's PFS consumer survey – number and type of products taken, overall distribution and by gender and age group.

		Total)		Gender				Age group			
		(n = 2359)		Male (n = 1141)		Female (n = 1218)		18–59 years (n = 1764)		≥60 years (n = 595)	
		n	% (95% CI)	n	% (95% CI)	n	% (95% CI)	n	% (95% CI)	n	% (95% CI)
Number of products taken	1 product	1975	83.7 (82.2–85.2)	980	85.9 (83.9–87.9)	995	81.7 (79.5–83.9)	1496	84.8 (83.1–86.5)	479	80.5 (77.3–83.7)
	2 products	289	12.3 (10.9–13.6)	123	10.8 (9.0–12.6)	166	13.6 (11.7–15.6)	196	11.1 (9.6–12.6)	93	15.6 (12.7–18.6)
	>2 products	95	4.0 (3.2–4.8)	38	3.3 (2.3–4.4)	57	4.7 (3.5–5.9)	72	4.1 (3.2–5.0)	23	3.9 (2.3–5.4)
Product type	1 single-botanical	1214	51.5 (49.5–53.5)	606	53.1 (50.2–56.0)	608	49.9 (47.1–52.7)	900	51.0 (48.7–53.4)	314	52.8 (48.8–56.8)
	1 multi -botanical	761	32.3 (30.4–34.2)	374	32.8 (30.1–35.5)	387	31.8 (29.2–34.4)	596	33.8 (31.6–36.0)	165	27.7 (24.1–31.3)
	2 or more single-botanical	104	4.4 (3.6–5.2)	45	3.9 (2.8–5.1)	59	4.8 (3.6–6.1)	72	4.1 (3.2–5.0)	32	5.4 (3.6–7.2)
	2 or more single- and multi-botanical	280	11.9 (10.6–13.2)	116	10.2 (8.4–11.9)	164	13.5 (11.6–15.4)	196	11.1 (9.6–12.6)	84	14.1 (11.3–16.9)

**Table 9:** PlantLIBRA's PFS consumer survey – number and type of products taken, by country.

		Finland (n = 401)		Germany (n = 398)		Italy (n = 378)		Romania (n = 400)		Spain (n = 402)		United Kingdom (n = 380)	
		n	% (95% CI)	n	% (95% CI)	n	% (95% CI)	n	% (95% CI)	n	% (95% CI)	n	% (95% CI)
Number of products taken	1 product	240	59.9 (55.1–64.7)	351	88.2 (85.0–91.4)	341	90.2 (87.2–93.2)	350	87.5 (84.3–90.8)	345	85.8 (82.4–89.2)	348	91.6 (88.8–94.4)
	2 products	93	23.2 (19.1–27.3)	45	11.3 (8.2–14.4)	34	9.0 (6.1–11.9)	40	10.0 (7.1–12.9)	48	11.9 (8.8–15.1)	29	7.6 (5.0–10.3)
	>2 products	68	17.0 (13.3–20.6)	2	0.5 (0.0–1.2)	3	0.8 (0.0–1.7)	10	2.5 (1.0–4.0)	9	2.2 (0.8–3.7)	3	0.8 (0.0–1.7)
Product type	1 single-botanical	82	20.5 (16.5–24.4)	172	43.2 (38.3–48.1)	176	46.6 (41.5–51.6)	251	62.8 (58.0–67.5)	212	52.7 (47.9–57.6)	321	84.5 (80.8–88.1)
	1 multi -botanical	158	39.4 (34.6–44.2)	179	45.0 (40.1–49.9)	165	43.7 (38.6–48.7)	99	24.8 (20.5–29.0)	133	33.1 (28.5–37.7)	27	7.1 (4.5–9.7)
	2 or more single-botanical	8	2.0 (0.6–3.4)	12	3.0 (1.3–4.7)	13	3.4 (1.6–5.3)	20	5.0 (2.9–7.1)	26	6.5 (4.1–8.9)	25	6.6 (4.1–9.1)
	2 or more single- and multi-botanical	153	38.2 (33.4–42.9)	35	8.8 (6.0–11.6)	24	6.4 (3.9–8.8)	30	7.5 (4.92–10.1)	31	7.7 (5.1–10.3)	7	1.8 (0.5–3.2)

Overall 51.5% of consumers used a single-botanical product and 32.3% used one multi-botanical product (Table 8). There were no significant differences between males and females in this usage pattern, but consumers aged over 60 used less multi-botanical products than those aged 18–59 (27.7% and 33.8% respectively) (Table 8). Overall, fewer consumers reported using two or more single-botanical products (4.4%) and two or more single- and multi-botanical products (11.9%) (Table 8).

There were some significant differences across countries in the type of products consumed (Table 9). In the six countries, the values for single-botanical products range from 84.5% (the United Kingdom) to 20.5% (Finland). Usage of multi-botanical products was reported in all countries, with the lowest proportion (7.1%) reported in the United Kingdom (Table 9). The use of two or more single-botanical products was low in all countries as was the usage of two or more single- and multi-botanical products. Finland was an exception to the latter, with 38.2% of respondents taking multiple products (Table 9).

The most common dose forms used (Table 10) are capsules (38.3%) and pills/tablets/lozenges (36.8%). No significant difference was observed in relation to gender or age (Table 10). Across the six countries (Table 11), solid forms are generally most popular, although capsules were used less frequently in Romania (17.7%). Liquid forms were less common in the United Kingdom (8.2%) and Germany (9.9%), but more common in Finland (26.2%) and Italy (26.4%) (Table 11).

**Table 10:** PlantLIBRA's PFS consumer survey – PFS dose forms used, per product used by a respondent, overall and by gender and age group.

Dose forms	Total		Gender				Age group			
	(n = 2874)		Male (n = 1358)		Female (n = 1516)		18–59 years (n = 2131)		≥60 years (n = 743)	
	n	% (95% CI)	n	% (95% CI)	n	% (95% CI)	n	% (95% CI)	n	% (95% CI)
Capsules^a^	1101	38.3 (36.5–40.1)	522	38.4 (35.9–41.0)	579	38.2 (35.8–40.6)	844	39.6 (37.5–41.7)	257	34.6 (31.2–38.0)
Pills/tablets/lozenges	1057	36.8 (35.0–38.5)	498	36.7 (34.1–39.2)	559	36.9 (34.4–39.3)	765	35.9 (33.8–37.9)	292	39.3 (35.8–42.8)
Liquid^b^	513	17.9 (16.5–19.3)	238	17.5 (15.5–19.6)	275	18.1 (16.2–20.1)	374	17.6 (15.9–19.2)	139	18.7 (15.9–21.5)
Ampoules	104	3.6 (2.9–4.3)	53	3.9 (2.9–4.9)	51	3.4 (2.5–4.3)	75	3.5 (2.7–4.3)	29	3.9 (2.5–5.3)
Other^c^	99	3.4 (2.8–4.1)	47	3.5 (2.5–4.4)	52	3.4 (2.5–4.4)	73	3.4 (2.7–4.2)	26	3.5 (2.2–4.8)

***Question asked***. And in which form do you usually take it? (mark the applicable form). *Possible responses*: Pills/tablets/lozenges; Softgel capsules/pearls; Hard capsules; Liquid (extract/syrup/drops); Sachets/packets; Ampoules; Other (specify); Not sure.

a Capsules: s°ftgels/pearls/hard capsules.

b Liquid: extract/syrups/dr°ps.

c Other: P°wders, Sachets/Packets, Bars and “Not sure”.

**Table 11:** PlantLIBRA's PFS consumer survey – PFS dose forms, per product used by a respondent, by country.

Dose forms	Finland (n = 665)		Germany (n = 446)		Italy (n = 417)		Romania (n = 464)		Spain (n = 465)		United Kingdom (n = 417)	
	n	% (95% CI)	n	% (95% CI)	n	% (95% CI)	n	% (95% CI)	n	% (95% CI)	n	% (95% CI)
Capsules^a^	206	31.0 (27.5–34.5)	225	50.5 (45.8–55.1)	144	34.5 (30.0–39.1)	82	17.7 (14.2–21.2)	250	53.8 (49.2–58.3)	194	46.5 (41.7–51.3)
Pills/tablets/lozenges	261	39.3 (35.5–43.0)	154	34.5 (30.1–39.0)	126	30.2 (25.8–34.6)	234	50.4 (45.9–55.0)	98	21.1 (17.4–24.8)	184	44.1 (39.4–48.9)
Liquid^b^	174	26.2 (22.8–29.5)	44	9.9 (7.1–12.6)	110	26.4 (22.1–30.6)	82	17.7 (14.2-21.2)	69	14.8 (11.6–18.1)	34	8.2 (5.5–10.8)
Ampoules	0	-	0	-	13	3.1 (1.5–4.8)	47	10.1 (7.4–12.9)	44	9.5 (6.8–12.1)	0	–
Other^c^	24	3.6 (2.2–5.0)	23	5.2 (3.1–7.2)	24	5.8 (3.5–8.0)	19	4.1 (2.3–5.9)	4	0.9 (0.1–1.7)	5	1.2 (0.2–2.2)

***Question asked***. And in which form do you usually take it? (mark the applicable form). *Possible responses*: Pills/tablets/lozenges; Softgel capsules/pearls; Hard capsules; Liquid (extract/syrup/drops); Sachets/packets; Ampoules; Other (specify); Not sure.

a Capsules: softgels/pearls/hard capsules.

b Liquid: extract/syrups/drops.

c Other: Powders, Sachets/Packets, Bars and “Not sure”.

### Botanicals used

A total of 491 botanicals -used in at least one PFS- were reported across the six participating countries. An overview of all the reported botanicals -clustered by intervals of frequency of intake (number of consumers ranging from 194 to 5)- is shown in Table 12. Based on the survey results, the eleven most frequently used botanicals (numbers of consumers ranging from 194 to 100) in descending order are *Ginkgo biloba* (ginkgo), *Oenothera biennis* (evening primrose), *Cynara scolymus* (artichoke), *Panax ginseng* (ginseng), *Aloe vera* (aloe), *Foeniculum vulgare* (fennel), *Valeriana officinalis* (valerian), *Glycine max* (soybean), *Melissa officinalis* (lemon balm), *Echinacea purpurea* (echinacea) and *Vaccinium myrtillus* (blueberry) (Table 12).

**Table 12:** PlantLIBRA's PFS consumer survey – botanicals used by at least 5 respondents, ordered by the "n of respondents”.

Used by n≥75 respondents		Used by n≥40-<75 respondents		Used by n ≥20-<40 respondents		Used by n≥5-<20 respondents	
n	Botanical(s)	n	Botanical(s)	n	Botanical(s)	n	Botanical(s)
194	*Ginkgo biloba; Oenothera biennis*	74	*Glycyrrhiza glabra*	38	*Cichorium intybus; Malus pumila*	19	*Achillea millefolium; Arctium lappa; Centella asiatica; Punica granatum; Raphanus sativus; Pyrus communis*
177	*Cynara scolymus*	72	*Mentha piperita; Paullinia cupana*	37	*Curcuma longa*	18	*Artemisia absinthium; Pollen; Lecithin*
170	*Panax ginseng*	71	*Malpighia glabra*	36	*Ananas comosus*	17	*Betula pubescens; Spirulina spec.; Vegetable charcoal;*
145	*Aloe vera*	70	*Oenothera spec.*	35	*Daucus carota; Glycine spec.*	16	*Origanum majorana; Ruscus aculeatus;Terminalia chebula*
131	*Foeniculum vulgare ssp*	69	*Silybum marianum*	34	*Myristica fragrans*	15	*Citrus paradise; Eschscholzia californica; Medicago sativa; Picea spec.; Vaccinium oxycoccus; Inulin*
128	*Valeriana officinalis*	66	*Citrus limon; Matricaria chamomilla*	33	*Crataegus monogyna; Cucurbita spec.; Dianthus spec.; Monascus purpureus*	14	*Althaea officinalis; Cuminum cyminum; Eryngium planum; Laminaria digitata; Rhamnus purshianus; Trigonella foenum-graecum; Zea mays*
103	*Glycine max; Melissa officinalis*	64	*Urtica dioica*	32	*Petroselinum crispum; Vaccinium macrocarpon*	13	*Chelidonium majus; Dioscorea villosa; Gossypium spec.; Hyssopus officinalis; Lactuca sativa; Origanum vulgare; Orthosiphon stamineus; Piper nigrum; Theobroma cacao; Trifolium pratense; Uncaria tomentosa; Lycopene; Equisetum spec.; Valeriana spec.*
102	*Echinacea purpurea*	63	*Thymus vulgaris*	31	*Coriandrum sativum; Echinaca spec.; Elettaria cardamomum; Prunus domestica*	12	*Asparagus officinalis; Azadirachta indica; Cassia occidentalis; Eucalyptus globulus; Tagetes erecta; Mentha spec.; Smilax officinalis; Xanthium spinosum*
100	*Vaccinium myrtillus;*	61	*Salvia officinalis*	30	*Cymbopogon citratus; Rhodiola rosea;*	11	*Abies alba; Artemisia abrotanum; Cetraria islandica; Cinnamomum camphora; Ilex paraguariensis; Laurus nobilis; Nasturtium officinale; Salix alba; Tilia spec.; Fraxinus excelsior; Gentiana asclepiadea; Triticum aestivum*
89	*Camellia sinensis; Zingiber officinale*	60	*Cassia senna; Rosmarinus officinalis*	29	*Calendula officinalis*	10	*Aegle marmelos; Aquilegia spec.; Armoracia rusticana; Brassica oleracea ssp.; Cheilocostus speciosus; Kaempferia galangal; Lepidium meyenii; Pimenta dioica; Populus nigra; Potentilla aurea; Santalum spec.; Sida cordifolia; Terminalia arjuna; Thymus serpyllum; Rubus fruticosus; Carlina acaulis; Centaurium spec.; Ganoderma lucidum; Tamarix gallica; Ceratonia siliqua*
88	*Pimpinella anisum*	59	*Hypericum perforatum; Lavandula angustifolia*	28	*Eleutherococcus senticosus; Fucus vesiculosus; Plantago ovate; Solanum lycopersicum; Spirulina platensis; Saccharomyces cerevisiae*	9	*Aesculus hippocastanum; Aloe ferox; Berberis aristata; Brassica oleracea var. botrytis; Capparis spinosa; Capsicum annuum var. annuum; Hieracium pilosella; Opuntia ficus-indica; Serenoa repens; Solanum nigrum; Tribulus terrestris; Melissa spec.*
87	*Vitis vinifera*	58	*Carum carvi*	27	*Citrus aurantium*	8	*Allium cepa; Apium graveolens; Boswellia serrate; Coffea spec.; Euterpe oleracea; Fumaria officinalis; Griffonia simplicifolia; Illicium verum; Malva sylvestris; Prunus armeniaca; Raphanus sativus convar. Sativus; Solidago virgaurea; Tamarindus indica; Carotene; Garcinia cambogia; Soy lecithin*
81	*Taraxacum officinale*	53	*Ribes nigrum*	26	*Schisandra chinensis; Flavonoids; Syzygium aromaticum*	7	*Acorus calamus; Angelica sinensis; Ascophyllum nodosum; Elymus repens; Ficus carica; Hamamelis virginiana; Phaseolus vulgaris; Prunus persica; Rheum spec.; Lutein; Capsicum annuum; Fraxinus spec.; Chamomile Eng; Violeta tricolor;*
79	*Echinacea angustifolia*	52	*Oryza sativa;*	25	*Angelica archangelica; Beta vulgaris ssp.vulgaris var. conditiva; Citrus sinensis; Juniperus communis; Peumus boldus*	6	*Brassica nigra; Brassica oleracea convar. acephala; Capsicum frutescens; Carthamus tinctorius; Cordyceps sinensis; Dioscorea spec.; Drosera rotundifolia; Echinacea pallida; Emblica officinalis; Fallopia japonica; Hedera spec.; Nigella sativa; Plantago psyllium; Satureja hortensis; Tilia platyphyllos; Hibiscus rosa-sinensis; Cirsium spec.; Fragaria spec.; Viola tricolor; Lavandula spec.; Fructooligosaccharides*
78	*Allium sativum Passiflora incarnata;*	48	*Hippophae rhamnoides*	23	*Borago officinalis; Gentiana lutea; Helianthus annuus; Ocimum basilicum; Panicum miliaceum; Pinus spec.*	5	*Aloe spec.; Alpinia galanga; Chamaemelum nobile; Coffea arabica; Cola acuminata; Cyamopsis tetragonoloba; Equisetum telmateia; Fagopyrum esculentum; Hibiscus sabdariffa; Pinus pinaster; Pinus sylvestris; Thymus spec.; Undaria pinnatifida; Withania somnifera; Isoflavones; Arecaceae spec.; Fallopia multiflora*
77	*Linum usitatissimum*	46	*Triticum spec.*	22	*Plantago lanceolata; Rhamnus frangula; Vaccinium vitis-idaea*		
76	*Equisetum arvense*	43	*Rosa canina; Cinnamomum spec.*	21	*Carica papaya; Cinnamomum verum; Crataegus spec.; Hordeum vulgare; Polygonum aviculare; Saccharum officinarum; Spinacia oleracea*		
75	*Harpagophytum procumbens; Olea europaea*	42	*Sambucus nigra*	20	*Algae; Avena sativa; Betula spec.; Fiilipendula ulmaria; Humulus lupulus*		

Table 13 shows the overall unweighted ranking of botanicals, 1–40, according to the number of consumers, in decreasing order. Table 13 also shows that when unweighted overall data are stratified by gender, only slight differences between men and women become evident and only *Glycine max* (soybean) was used significantly more by women than by men (Table 13).

**Table 13:** PlantLIBRA's PFS consumer survey – distribution of the overall top-40 botanicals' reported consumption and the ranking of these botanicals when stratified by gender and age group.

	All consumers			Gender						Age group					
Botanicals				Male			Female			18-59 years			≥60 years		
	Rank^a^	n	% (95% CI)	Rank^b^	n	% (95% CI)	Rank^b^	n	% (95% CI)	Rank^b^	n	% (95% CI)	Rank^b^	n	% (95% CI)
*Ginkgo biloba*	1	194	8.2 (7.1–9.3)	1	107	9.4 (7.7–11.0)	3	87	7.1 (5.7–8.6)	2	135	7.7 (6.4–8.9)	1	59	9.9 (7.5–12.3)
*Oenothera biennis*	2	194	8.2 (7.1–9.3)	3	85	7.5 (5.9–8.9)	1	109	9.0 (7.4–10.5)	1	145	8.2 (6.9–9.5)	2	49	8.2 (6.0–10.4)
*Cynara scolymus*	3	173	7.3 (6.3–8.4)	5	73	6.4 (5.0–7.8)	2	100	8.2 (6.7–9.7)	4	128	7.3 (6.1–8.4)	4	45	7.6 (5.4–9.6)
*Panax ginseng*	4	167	7.1 (6.0–8.1)	2	94	8.2 (6.6–9.8)	5	73	6.0 (4.7–7.3)	3	133	7.5 (6.3–8.7)	6	34	5.7 (3.9–7.5)
*Aloe vera*	5	145	6.2 (5.2–7.1)	4	80	7.0 (5.5–8.5)	7	65	5.3 (4.1–6.6)	5	99	5.6 (4.5–6.7)	3	46	7.7 (5.6–9.8)
*Foeniculum vulgare ssp.*	6	132	5.6 (4.7–6.5)	7	59	5.2 (3.9–6.4)	4	73	6.0 (4.7–7.3)	6	99	5.6 (4.5–6.7)	7	33	5.6 (3.7–7.3)
*Valeriana officinalis*	7	125	5.3 (4.4–6.2)	6	62	5.4 (4.1–6.7)	8	63	5.2 (3.9–6.4)	7	97	5.5 (4.4–6.5)	9	28	4.7 (3.0–6.4
*Glycine max*	8	103	4.4 (3.5–5.2)	24	34	3.0 (2.0–3.9)	6	69	5.7 (4.4–6.9)	10	81	4.6 (3.6–5.5)	14	22	3.7 (2.2–5.2)
*Melissa officinalis*	9	103	4.4 (3.5–5.2)	8	53	4.7 (3.4–5.8)	10	50	4.1 (3.0–5.2)	9	82	4.7 (3.7–5.6)	17	21	3.5 (2.1–5.0)
*Echinacea purpurea*	10	102	4.3 (3.5–5.1)	12	43	3.8 (2.7–4.8)	9	59	4.8 (3.6–6.0)	8	83	4.7 (3.7–5.7)	21	19	3.2 (1.8–4.6)
*Vaccinium myrtillus*	11	100	4.2 (3.4–5.1)	9	53	4.7 (3.4–5.8)	13	47	3.9 (2.8–4.9)	12	71	4.0 (3.1–4.9)	8	29	4.9 (3.1–6.6)
*Pimpinella anisum*	12	89	3.8 (3.0–4.5)	11	47	4.1 (3.0–5.2)	21	42	3.5 (2.4–4.4)	16	65	3.7 (2.8–4.5)	11	24	4.0 (2.5–5.6)
*Zingiber officinale*	13	89	3.8 (3.0–4.5)	10	53	4.7 (3.4–5.8)	29	36	3.0 (2.0–3.9)	15	66	3.7 (2.9–4.6)	13	23	3.9 (2.3–5.4)
*Camellia sinensis*	14	87	3.7 (2.9–4.5)	17	39	3.4 (2.4–4.4)	11	48	3.9 (2.9–5.0)	11	72	4.1 (3.2–5.0)	33	15	2.5 (1.3–3.7)
*Vitis vinifera*	15	87	3.7 (2.9–4.5)	16	41	3.6 (2.5–4.6)	15	46	3.8 (2.7–4.8)	13	71	4.0 (3.1–4.9)	32	16	2.7 (1.4–4.0)
*Taraxacum officinale*	16	80	3.4 (2.7–4.1)	21	36	3.2 (2.1–4.1)	17	44	3.6 (2.6–4.6)	17	65	3.7 (2.8–4.5)	34	15	2.5 (1.3–3.7)
*Echinacea angustifolia*	17	79	3.4 (2.6–4.1)	23	34	3.0 (2.0–3.9)	16	45	3.7 (2.6–4.7)	20	60	3.4 (2.6–4.2)	20	19	3.2 (1.8–4.6)
*Passiflora incarnata*	18	78	3.3 (2.6–4.0)	30	30	2.6 (1.7–3.5)	12	48	3.9 (2.9–5.0)	19	61	3.5 (2.6–4.3)	30	17	2.9 (1.5–4.2)
*Linum usitatissimum*	19	77	3.3 (2.6–4.0)	13	43	3.8 (2.7–4.8)	33	34	2.8 (1.9–3.7)	22	56	3.2 (2.4–4.0)	16	21	3.5 (2.1–5.0)
*Equisetum arvense*	20	76	3.2 (2.5–3.9)	19	37	3.2 (2.2–4.2)	23	39	3.2 (2.2–4.2)	23	55	3.1 (2.3–3.9)	15	21	3.5 (2.1–5.0)
*Allium sativum*	21	75	3.2 (2.5–3.9)	28	32	2.8 (1.9–3.7)	18	43	3.5 (2.5–4.5)	29	50	2.8 (2.1–3.6)	10	25	4.2 (2.6–5.8)
*Harpagophytum procumbens*	22	75	3.2 (2.5–3.9)	18	39	3.4 (2.4–4.4)	26	36	3.0 (2.0–3.9)	40	40	2.3 (1.6–2.9)	5	35	5.9 (4.0–7.7)
*Olea europaea*	23	75	3.2 (2.5–3.9)	27	33	2.9 (1.9–3.8)	20	42	3.5 (2.4–4.4)	24	55	3.1 (2.3–3.9)	19	20	3.4 (1.9–4.8)
*Glycyrrhiza glabra*	24	74	3.1 (2.4–3.8)	26	33	2.9 (1.9–3.8)	22	41	3.4 (2.4–4.4)	25	54	3.1 (2.3–3.8)	18	20	3.4 (1.9–4.8)
*Mentha piperita*	25	72	3.1 (2.4–3.8)	20	36	3.2 (2.1–4.1)	27	36	3.0 (2.0–3.9)	27	53	3.0 (2.2–3.8)	22	19	3.2 (1.8–4.6)
*Paullinia cupana*	26	72	3.1 (2.4–3.8)	14	43	3.8 (2.7–4.8)	38	29	2.4 (1.5–3.2)	14	66	3.7 (2.9–4.6)	74	6	1.0 (0.2–1.8)
*Malpighia glabra*	27	71	3.0 (2.3–3.7)	15	41	3.6 (2.5–4.6)	37	30	2.5 (1.6–3.3)	18	61	3.5 (2.6–4.3)	51	10	1.7 (0.7–2.7)
*Oenothera spec*	28	70	3.0 (2.3–3.7)	41	23	2.0 (1.2–2.8)	14	47	3.9 (2.8–4.9)	21	59	3.3 (2.5–4.2)	47	11	1.9 (0.8–2.9)
*Silybum marianum*	29	69	2.9 (2.2–3.6)	25	34	3.0 (2.0–3.9)	30	35	2.9 (1.9–3.8)	32	46	2.6 (1.9–3.3)	12	23	3.9 (2.3–5.4)
*Matricaria chamomilla*	30	67	2.8 (2.2–3.5)	34	29	2.5 (1.6–3.4)	25	38	3.1 (2.1–4.1)	26	54	3.1 (2.3–3.8)	38	13	2.2 (1.0–3.3)
*Citrus limon*	31	66	2.8 (2.1–3.5)	37	24	2.1 (1.3–2.9)	19	42	3.5 (2.4–4.4)	30	48	2.7 (2.0–3.5)	25	18	3.0 (1.7–4.4)
*Urtica dioica*	32	64	2.7 (2.1–3.4)	31	30	2.6 (1.7–3.5)	34	34	2.8 (1.9–3.7)	28	51	2.9 (2.1–3.7)	37	13	2.2 (1.0–3.3)
*Thymus vulgaris*	33	63	2.7 (2.0–3.3)	36	28	2.5 (1.6–3.3)	31	35	2.9 (1.9–3.8)	33	44	2.5 (1.8–3.2)	24	19	3.2 (1.8–4.6)
*Salvia officinalis*	34	61	2.6 (2.0–3.2)	32	22	1.9 (1.1–2.7)	35	39	3.2 (2.2–4.2)	34	43	2.4 (1.7–3.1)	29	18	3.0 (1.7–4.4)
*Cassia senna*	35	60	2.5 (1.9–3.2)	43	29	2.5 (1.6–3.4)	24	31	2.6 (1.7–3.4)	37	43	2.4 (1.7–3.1)	28	17	2.9 (1.5–4.2)
*Rosmarinus officinalis*	36	60	2.5 (1.9–3.2)	38	24	2.1 (1.3–2.9)	28	36	3.0 (2.0–3.9)	39	41	2.3 (1.6–3.0)	23	19	3.2 (1.8–4.6)
*Carum carvi*	37	59	2.5 (1.9–3.1)	22	35	3.1 (2.1–4.0)	43	24	2.0 (1.2–2.7)	31	46	2.6 (1.9–3.3)	36	13	2.2 (1.0–3.3)
*Hypericum perforatum*	38	59	2.5 (1.9–3.1)	29	31	2.7 (1.8–3.6)	39	28	2.3 (1.5–3.1)	35	43	2.4 (1.7–3.1)	31	16	2.7 (1.4–4.0)
*Lavandula angustifolia*	39	57	2.4 (1.8–3.0)	40	23	2.0 (1.2–2.8)	32	34	2.8 (1.9–3.7)	36	43	2.4 (1.7–3.1)	35	14	2.4 (1.1–3.5)
*Ribes nigrum*	40	53	2.3 (1.7–2.8)	42	22	1.9 (1.1–2.7)	36	31	2.6 (1.7–3.4)	38	41	2.3 (1.6–3.0)	41	12	2.0 (0.9–3.1)

a Products ordered according to the consumer distribution of the overall top-40 used botanicals (unweighted ranking).

b Ranks show the shifts of the botanicals in the position of the overall 1–40 unweighted ranking when stratified by gender and age group.

When the overall top-40 botanical data are stratified by age groups, slight differences become evident. In the group of 18–59 year-olds, the most frequently used botanicals comply with the overall data just differing in the ranking, with *Oenothera biennis* (evening primrose) being the most frequently used botanical (Table 13). In the group of 60+ year-old a stronger shift can be observed (Table 13). Although *Ginkgo biloba* (ginkgo) is still the most reported botanical -as in the overall ranking- other botanicals are frequently used by that age group. *Harpagophytum procumbens* (devil's claw), *Vaccinium myrtillus* (blueberry) and *Allium sativum* (garlic) are within the most frequently reported botanicals, whereas *Glycine max* (soybean), *Melissa officinalis* (lemon balm) and *Echinacea purpurea* (echinacea) do not appear in the top 10 ranking.

Cross-country differences emerge when considering the overall top-40 botanicals more frequently present in PFS products in each of the individual six countries (Table 14). In the Finnish sample, products containing *Glycine max* (soybean) are the most frequently used, followed by those containing *Echinacea angustifolia and purpurea* (echinacea). German consumers reported *Ginkgo biloba* (ginkgo), *Cynara scolymus* (artichoke) and *Olea europea* (olive) as the most frequently used botanicals; whilst in Romania, *Ginkgo biloba* (ginkgo) was also the ingredient most frequently indicated, followed by *Aloe vera* (aloe) and *Panax ginseng* (ginseng). Amongst Italian consumers, *Aloe vera* (aloe) was the most frequently used botanical, followed by *Foeniculum vulgare* (fennel) and *Valeriana officinalis* (valerian). In Spain, PFS containing *Cynara scolymus* (artichoke) were the most frequently used products, followed by those containing *Valeriana officinalis* (valerian) and *Equisetum arvense* (horsetail). In the United Kingdom, *Oenothera biennis* (evening primrose) was by far the most frequently reported botanical ingredient, followed by *Panax ginseng* (ginseng) and *Hypericum perforatum* (St. John's wort). In addition, there is a great variation in the ranking of consumed botanicals among countries.

**Table 14:** PlantLIBRA's PFS consumer survey – ranking of the overall top-40 botanicals' reported consumption when stratified by country.

Botanicals	Finland			Germany			Italy			Romania			Spain			United Kingdom		
	Rank^a^	n	% (95% CI)	Rank^a^	n	% (95% CI)	Rank^a^	n	% (95% CI)	Rank^a^	n	% (95% CI)	Rank^1^	n	% (95% CI)	Rank^a^	n	% (95% CI)
*Ginkgo biloba*		0	-	1	50	12.6 (9.3–15.8)	12	17	4.5 (2.4–6.6)	1	105	26.3 (21.9–30.6)	27	11	2.7 (1.1–4.3)	11	11	2.9 (1.2–4.6)
*Oenothera biennis*		0	-	22	15	3.8 (1.9–5.6)	174	1	0.3 (0.0–0.8)	164	1	0.3 (0.0–0.7)	20	13	3.2 (1.5–5.0)	1	164	43.2 (38.2–48.1)
*Cynara scolymus*	53	12	3.0 (1.3–4.7)	2	47	11.8 (8.6–15.0)	10	20	5.3 (3.0–7.6)	7	27	6.8 (4.3–9.2)	1	67	16.7 (13.0–20.3)		0	–
*Panax ginseng*	42	16	4.0 (2.1–5.9)	7	26	6.5 (4.1–9.0)	4	28	7.4 (4.8–10.1)	3	41	10.3 (7.3–13.2)	16	15	3.7 (1.9-5.6)	2	41	10.8 (7.7–13.9)
*Aloe vera*	172	1	0.3 (0.0–0.7)	25	12	3.0 (1.3–4.7)	1	44	11.6 (8.4–14.9)	2	47	11.8 (8.6–14.9)	37	8	2.0 (0.6–3.4)	4	33	8.7 (5.9–11.5)
*Foeniculum vulgare ssp.*	31	21	5.2 (3.1–7.4)	11	20	5.0 (2.9–7.2)	2	29	7.7 (5.0–10.4)	8	27	6.8 (4.3–9.2)	4	34	8.5 (5.7–11.2)	33	1	0.3 (0.0–0.8)
*Valeriana officinalis*	192	1	0.3 (0.0–0.7)	19	16	4.0 (2.1–6.0)	3	29	7.7 (5.0–10.4)	43	11	2.8 (1.2–4.4)	2	51	12.7 (9.4–15.9)	6	17	4.5 (2.4–6.6)
*Glycine max*	1	73	18.2 (14.4–22.0)	6	27	6.8 (4.3–9.3)	161	1	0.3 (0.0–0.8)		0	-	114	2	0.5 (0.0–1.2)		0	-
*Melissa officinalis*	14	39	9.7 (6.8–12.6)	12	20	5.0 (2.9–7.2)	7	25	6.6 (4.1–9.1)	74	5	1.3 (0.2–2.3)	18	14	3.5 (1.7–5.3)		0	-
*Echinacea purpurea*	3	55	13.7 (10.3–17.1)		0	-	59	5	1.3 (0.2–2.5)	13	24	6.0 (3.7–8.3)	70	4	1.0 (0.0–2.0)	7	14	3.7 (1.8–5.6)
*Vaccinium myrtillus*	23	30	7.5 (4.9–10.1)	30	12	3.0 (1.3–4.7)	5	28	7.4 (4.8–10.1)	15	20	5.0 (2.9–7.1)	43	8	2.0 (0.6–3.4)	26	2	0.5 (0.0–1.3)
*Pimpinella anisum*	16	36	9.0 (6.2–11.8)	28	12	3.0 (1.3–4.7)	38	8	2.1 (0.7–3.6)	21	15	3.8 (1.9–5.6)	11	18	4.5 (2.5–6.5)		0	–
*Zingiber officinale*	13	41	10.2 (7.3–13.2)	36	11	2.8 (1.2–4.4)	67	5	1.3 (0.2–2.5)	4	30	7.5 (4.9–10.1)	131	2	0.5 (0.0–1.2)		0	–
*Camellia sinensis*	28	23	5.7 (3.5–8.0)	16	16	4.0 (2.1–6.0)	22	12	3.2 (1.4–4.9)	47	10	2.5 (1.0–4.0)	6	26	6.5 (4.1–8.9)		0	–
*Vitis vinifera*	34	20	5.0 (2.9–7.1)	5	28	7.0 (4.5–9.6)	28	11	2.9 (1.2–4.6)	127	2	0.5 (0.0–1.2)	12	18	4.5 (2.5–6.5)	13	8	2.1 (0.7–3.6)
*Taraxacum officinale*	65	10	2.5 (1.0–4.0)	52	10	2.5 (1.0–4.1)	9	21	5.6 (3.2–7.9)	24	15	3.8 (1.9–5.6)	8	24	6.0 (3.7–8.3)		0	–
*Echinacea angustifolia*	2	55	13.7 (10.3–17.1)		0	–	48	6	1.6 (0.3–2.9)	117	2	0.5 (0.0–1.2)	31	10	2.5 (1.0–4.0)	15	6	1.6 (0.3–2.8)
*Passiflora incarnata*	75	8	2.0 (0.6–3.4)	62	7	1.8 (0.5–3.1)	6	26	6.9 (4.3–9.4)	65	7	1.8 (0.5–3.0)	5	30	7.5 (4.9–10.0)		0	–
*Linum usitatissimum*	24	28	7.0 (4.5–9.5)	27	12	3.0 (1.3–)4.7	95	3	0.8 (0.0–1.7)	14	24	6.0 (3.7–8.3)	73	4	1.0 (0.0–2.0)	16	6	1.6 (0.3–2.8)
*Equisetum arvense*	26	26	6.5 (4.1–8.9)	153	1	0.3 (0.0–0.7)	60	5	1.3 (0.2–2.5)	82	4	1.0 (0.0–2.0)	3	40	10.0 (7.0–12.9)		0	–
*Allium sativum*	27	25	6.2 (3.9–8.6)	92	3	0.8 (0.0–1.6)	69	4	1.1 (0.0–2.1)	64	7	1.8 (0.5–3.0)	7	24	6.0 (3.7–8.3)	10	12	3.2 (1.4–4.9)
*Harpagophytum procumbens*		0	–	9	21	5.3 (3.1–7.5)	20	13	3.4 (1.6–5.3)	55	9	2.3 (0.8–3.7)	40	8	2.0 (0.6–3.4)	5	24	6.3 (3.9–8.8)
*Olea europaea*	30	22	5.5 (3.3–7.7)	3	40	10.1 (7.1–13.0)		0	–	84	4	1.0 (0.0–2.0)	42	8	2.0 (0.6–3.4)	36	1	0.3 (0.0–0.8)
*Glycyrrhiza glabra*	47	14	3.5 (1.7–5.3)	18	16	4.0 (2.1–6.0)	17	14	3.7 (1.8–5.6)	10	26	6.5 (4.1–8.9)	71	4	1.0 (0.0–2.0)		0	–
*Mentha piperita*	4	47	11.7 (8.6–14.9)	24	14	3.5 (1.7–5.3)	78	4	1.1 (0.0–2.1)	75	5	1.3 (0.2–2.3)	119	2	0.5 (0.0–1.2)		0	–
*Paullinia cupana*	130	4	1.0 (0.0–2.0)	10	21	5.3 (3.1–7.5)	8	23	6.1 (3.7–8.5)	76	5	1.3 (0.2–2.3)	14	16	4.0 (2.1–5.9)	21	3	0.8 (0.0–1.7)
*Malpighia glabra*	12	41	10.2 (7.3–13.2)	21	15	3.8 (1.9–5.6)	18	14	3.7 (1.8–5.6)		0	–	169	1	0.3 (0.0–0.7)		0	–
*Oenothera spec*	10	43	10.7 (7.7–13.8)		0	–		0	–		0	–	10	20	5.0 (2.9–7.1)	14	7	1.8 (0.5–3.2)
*Silybum marianum*	190	1	0.3 (0.0–0.7)	35	11	2.8 (1.2–4.4)	15	15	4.0 (2.0–5.9)	23	15	3.8 (1.9–5.6)	19	14	3.5 (1.7–5.3)	9	13	3.4 (1.6–5.3)
*Matricaria chamomilla*	66	10	2.5 (1.0–4.0)	38	11	2.8 (1.2–4.4)	35	9	2.4 (0.8–3.9)	20	16	4.0 (2.1–5.9)	9	21	5.2 (3.1–7.4)		0	–
*Citrus limon*	7	43	10.7 (7.7–13.8)	112	2	0.5 (0.0–1.2)	29	10	2.7 (1.0–4.3)	146	1	0.3 (0.0–0.7)	30	10	2.5 (1.0–4.0)		0	–
*Urtica dioica*	9	43	10.7 (7.7–13.8)	53	10	2.5 (1.0–4.1)	133	2	0.5 (0.0–1.3)	89	4	1.0 (0.0–2.0)	66	5	1.2 (0.2–2.3)		0	–
*Thymus vulgaris*	6	47	11.7 (8.6–14.9)	177	1	0.3 (0.0–0.7)	66	5	1.3 (0.2–2.5)	87	4	1.0 (0.0–2.0)	53	6	1.5 (0.3–2.7)		0	–
*Salvia officinalis*	8	43	10.7 (7.7–13.8)	80	5	1.3 (0.2–2.4)	82	4	1.1 (0.0–2.1)	66	7	1.8 (0.5–3.0)	124	2	0.5 (0.0–1.2)		0	–
*Cassia senna*		0	–		0	–	11	19	5.0 (2.8–7.2)	11	25	6.3 (3.9–8.6)	22	12	3.0 (1.3–4.7)	17	4	1.1 (0.0–2.1)
*Rosmarinus officinalis*	64	10	2.5 (1.0–4.0)	34	11	2.8 (1.2–4.4)	129	2	0.5 (0.0–1.3)	12	25	6.3 (3.9–8.6)	25	12	3.0 (1.3–4.7)		0	–
*Carum carvi*		0	–	8	23	5.8 (3.5–8.1)	33	9	2.4 (0.8–3.9)	9	26	6.5 (4.1–8.9)	149	1	0.3 (0.0–0.7)		0	–
*Hypericum perforatum*		0	–	157	1	0.3 (0.0–0.7)	34	9	2.4 (0.8–3.9)	56	9	2.3 (0.8–3.7)	63	5	1.2 (0.2–2.3)	3	35	9.2 (6.3–12.1)
*Lavandula angustifolia*	17	34	8.5 (5.8–11.2)	161	1	0.3 (0.0–0.7)		0	–	60	8	2.0 (0.6–3.4)	32	10	2.5 (1.0–4.0)	19	4	1.1 (0.0–2.1)
*Ribes nigrum*	20	32	8.0 (5.3–10.6)	172	1	0.3 (0.0–0.7)	44	7	1.9 (0.5–3.2)	176	1	0.3 (0.0–0.7)	24	12	3.0 (1.3–4.7)		0	–

a Ranks show the shifts of the botanicals in the position of the overall 1–40 unweighted ranking when stratified by country.

## Discussion

The present paper reports the findings from a European multi-country survey of PFS consumers: the PlantLIBRA PFS consumer survey. Data on the usage of PFS at the European level are limited, confined in the main to commercial market data [7] as opposed to consumer survey data, as evidenced in the recent review by Bishop and Lewith (2010)[4], where only 13% of population based consumption studies were in Europe. The European Food Safety Authority (EFSA) has recognised the lack of data in the sector and has published a number of reports addressing related issues [15]–[16].

To our knowledge this is the first survey of consumers of PFS undertaken in Europe. In total 2359 consumers of PFS were recruited in this cross-sectional retrospective survey. Across all countries prevalence of usage is estimated at 18.8%. Vargas-Murga and colleagues (2011)[9] highlighted that comparable data at European level is difficult to identify when reviewing prevalence data from a selected number of European studies, evaluating PFS or CAM usage, with values ranging from 0.8% to 70%. All studies were based on nationally representative samples but the definition of use of supplements varied widely, in some cases being self-defined by the participant and not distinguishing between PFS and HMP. The use of dietary supplements in a European population was measured in the European Prospective Investigation into Cancer and Nutrition (EPIC) study [8]. Usage was measured by completion of a standardised 24-hour dietary recall and included all dietary supplements that met the EU Directive 2002/46/EC. Results indicated significant differences in overall dietary supplement use between countries with herbs/plant-based supplements representing 8–17% of the products used across the ten countries.

The prevalence rate reported here can be compared to rates from surveys conducted in the United States, where data on usage of dietary supplements, including herbal supplements, is collected more routinely. It is similar to the rate reported in the 2002 and 2007 National Health Interview Surveys (NHIS), 18.9% and 17.9% respectively [20]; higher than the rates of both the Eisenberg's survey [21] and the Slone survey [22], with 14% and 12.1% respectively; and lower than the 2002 Health and Diet Survey (42%) [23] or the 1999 Kaiser Permanent Medical Care Program of Northern California (KPMCP), with a prevalence of 28.3% [24]. These differences in prevalence across studies may in part be due to the distinct selected population samples, survey methodologies (i.e. sampling methods, data collection techniques) or definitions of usage, as well as possible variations in health beliefs and health behaviour of the different populations of study [9], [24].

Survey respondents were recruited to set quotas for both age and gender to reflect characteristics previously reported for dietary supplement users. Age and gender are significant determinants of the consumption of dietary supplements in general and in botanical products in particular. Previous studies on the use of dietary supplements or other herbal-related use show a higher consumption among women as compared to men [1], [17], [24]–[28] and a higher consumption among older adults as compared to younger adults [24], [29]–[32].

Other characteristics of dietary supplements users that have been reported previously in the literature include having higher educational attainment and socioeconomic status [24], [33]–[34], being less likely to smoke [10], [32], [35], being more physically active [10], [29], [32]. Bailey et al. also reported a moderate alcohol consumption (1 drink per day) among dietary supplement users as compared to nonusers. In contrast, a study by Rovira et al. in a southern European population found no differences in lifestyle factors such as physical activity, smoking, and alcohol consumption between dietary supplement users and non-users [36]. Our survey population consists exclusively of PFS consumers, but their responses to a series of questions on health-related lifestyle factors reflect some of the characteristics mentioned above. The majority of PFS consumers perceived their health status to be “very good or good”, reflecting results reported in a number of studies on dietary supplement users [32] and CAM and dietary supplement users [24], where the answer “very good or excellent” has been reported for self-reported health status.

The survey results indicate that most consumers reported using one PFS product in the preceding 12 months, with 12% using two products and 4% using more than two. Individual country data show that Finnish consumers use more than one product and PFS with more than one botanical component, and the opposite is observed in the United Kingdom, where about 90% of the consumers use only one PFS and the products contain mostly only one botanical. In the United States, recent studies have reported that about half of the adults report using one or more dietary supplements [32], [37]. One of these studies also found that over half of dietary supplement consumers used a single-botanical product and one third used one multi-botanical product [32]. Similar results were found in our survey across all countries i.e. smaller numbers of consumers reported using two or more single-botanical products (4.4%) and two or more single- and multi-botanical products (11.9%).

A wide variety of botanicals (491) is used in PFS consumed by the respondents in this survey. Overall raw data show that the most frequently (n>100) used botanicals in descending order are *Ginkgo biloba* (ginkgo), *Oenothera biennis* (evening primrose), *Cynara scolymus* (artichoke), *Panax ginseng* (ginseng), *Aloe vera, Foeniculum vulgare* (fennel), *Valeriana officinalis* (valeriana), *Glycine max* (soybean), *Melissa officinalis* (lemon balm), *Echinacea purpurea* (echinacea) *and Vaccinium myrtillus* (blueberry). These results reflect some commercial data which reported that ginkgo followed by echinacea, garlic and ginseng were the four most commercially important botanicals in the combined markets of seventeen EC Member States. In this data, echinacea and ginkgo were part of the composition of products registered as medicines [7], [9], which were excluded from our survey. Similarly, the US Food and Drug Administration 2002 Health and Diet Survey, also a 12-month retrospective study, reported the same four herbs/botanicals/or other nonvitamin-nonmineral dietary supplements being the most used by its adult population – although in the following order: echinacea, garlic, ginkgo and ginseng (the latter including tea) [23]. Schaffer et al. also reported echinacea as the most consumed botanical in the Californian 1999 KPMCP survey, followed by ginkgo [24]. Differences between countries are more evident; the top list of botanicals contained in PFS for each single country complies little with the ranking of the overall data. As mentioned earlier, data were not weighted by country population size because of the study methodology which included very similar country-sample sizes of PFS consumers only, therefore caution is needed when drawing conclusions from these results at the overall 6-country level. Overall data merely describes the collected pooled data from all 6 countries. However, if the overall ranking data were to be weighted by the population size -for example the 1–5 ranking data-, the positions of the botanicals would have been only slightly altered, with *Oenothera biennis* (evening primrose) being the most consumed one, followed by *Cynara scolymus* (artichoke) *Ginkgo biloba* (ginkgo), *Panax ginseng* (ginseng) and *Aloe vera* (aloe).

The results of the survey highlight clear differences between countries in terms of the botanicals used by consumers as PFS. This may reflect the fact that the current legal and regulatory framework for botanicals has a major influence on the nature of the local PFS markets. The EU Directive 2002/46/EC does not provide a clear definition of what is encompassed by the term ‘other substance with a nutritional or physiological effect', although it is generally accepted that botanicals and their extracts fall into this category. Current legislation varies across Europe, with significant differences in the botanical species permitted in PFS. These issues were highlighted in a recent review of the regulations applicable to PFS in the European Union by Silano et al. [38]. They provide examples of the different national approaches for the use of selected botanicals in food supplements in the EU Member States.

To illustrate the above complexity, in Germany, food supplements are regulated by the German Regulation on Food Supplements [39] and the German Law on Food and Feed [40]. Positive lists are available for minerals and vitamins. Food supplements have to be registered with the Federal Office of Consumer Protection and Food Safety [41]. The BVL maintains a list of plants which are either classified as a food or a medicinal product, and which is neither considered complete nor legally binding [41]. Data on the intake of PFS in Germany is limited and, despite food supplement intake being recorded in recent health and nutrition surveys [42]–[44], no specific data was published on PFS intake. The results from the PlantLIBRA consumer survey do not include *Valeriana officinalis* in the German top list of botanicals used in PFS, whereas 1852 medicinal products containing Valerian exist on the market [40]. The absence of *Valeriana officinalis* in the German list of botanicals can be explained by its dominant presence as a HMP in the German market.

The results of this survey represent some of the first data on the usage of PFS at European level, thus addressing the existing deficit of such data by collecting retrospective data directly from consumers in six European countries. The benefits of the data collection instrument used in this study included that it was relatively straightforward to administer, did not alter habitual usage patterns and allowed the classification of individuals into categories of usage. However, the results must be considered in the light of their limitations. The sample population comprises exclusively of PFS consumers, recruited to meet very specific inclusion criteria and hence no comparisons can be made with the general population. Future studies should seek to compare users and non-users of PFS.

Further limitations relate to the retrospective nature of the data being collected. In many cases respondents needed to rely on memory to report usage of products in the preceding 12 months. Where products are available for inspection at data collection, there is a need for careful recording of product details to ensure accurate coding. The lack of a comprehensive product database containing reliable ingredient information meant a bespoke database needed to be created. Future studies should seek to collect prospective data. Prospective dietary intake surveys offer an ideal opportunity to collect data on supplement use in conjunction with data on food and beverages. Care needs to be taken to collect sufficiently detailed information about ingredients and amounts consumed. For example, in the US, the Alternative Health/CAM supplement of the National Health Interview Survey (NHIS) is part of an annual, nationally representative survey of US adults. It contains data on adults' use of 10 herbs most commonly taken to treat a specific health condition in the preceding 12 months [13]; the survey has a separate section on dietary supplements and distinguishes “natural herbs” from vitamins and minerals. The authors would like to encourage researchers to implement future surveys/studies which are necessary to overcome the bottlenecks in PFS risk and benefit assessments at the European level.
